# Extent of Risk-Aligned Surveillance for Cancer Recurrence Among Patients With Early-Stage Bladder Cancer

**DOI:** 10.1001/jamanetworkopen.2018.3442

**Published:** 2018-09-28

**Authors:** Florian R. Schroeck, Kristine E. Lynch, Ji won Chang, Todd A. MacKenzie, John D. Seigne, Douglas J. Robertson, Philip P. Goodney, Brenda Sirovich

**Affiliations:** 1 Norris Cotton Cancer Center, Dartmouth Hitchcock Medical Center, Lebanon, New Hampshire; 2 The Dartmouth Institute for Health Policy and Clinical Practice, Geisel School of Medicine at Dartmouth College, Lebanon, New Hampshire; 3 Section of Urology, Dartmouth Hitchcock Medical Center, Lebanon, New Hampshire; 4 White River Junction VA Medical Center, White River Junction, Vermont; 5 VA Salt Lake City Health Care System, Salt Lake City, Utah; 6 University of Utah, Salt Lake City

## Abstract

**Importance:**

Cancer care guidelines recommend aligning surveillance frequency with underlying cancer risk, ie, more frequent surveillance for patients at high vs low risk of cancer recurrence.

**Objective:**

To assess the extent to which such risk-aligned surveillance is practiced within US Department of Veterans Affairs facilities by classifying surveillance patterns for low- vs high-risk patients with early-stage bladder cancer.

**Design, Setting, and Participants:**

US national retrospective cohort study of a population-based sample of patients diagnosed with low-risk or high-risk early-stage bladder between January 1, 2005, and December 31, 2011, with follow-up through December 31, 2014. Analyses were performed March 2017 to April 2018. The study included all Veterans Affairs facilities (n = 85) where both low- and high-risk patients were treated.

**Exposures:**

Low-risk vs high-risk cancer status, based on definitions from the European Association of Urology risk stratification guidelines and on data extracted from diagnostic pathology reports via validated natural language processing algorithms.

**Main Outcomes and Measures:**

Adjusted cystoscopy frequency for low-risk and high-risk patients for each facility, estimated using multilevel modeling.

**Results:**

The study included 1278 low-risk and 2115 high-risk patients (median [interquartile range] age, 77 [71-82] years; 99% [3368 of 3393] male). Across facilities, the adjusted frequency of surveillance cystoscopy ranged from 3.7 to 6.2 (mean, 4.8) procedures over 2 years per patient for low-risk patients and from 4.6 to 6.0 (mean, 5.4) procedures over 2 years per patient for high-risk patients. In 70 of 85 facilities, surveillance was performed at a comparable frequency for low- and high-risk patients, differing by less than 1 cystoscopy over 2 years. Surveillance frequency among high-risk patients statistically significantly exceeded surveillance among low-risk patients at only 4 facilities. Across all facilities, surveillance frequencies for low- vs high-risk patients were moderately strongly correlated (*r* = 0.52; *P* < .001).

**Conclusions and Relevance:**

Patients with early-stage bladder cancer undergo cystoscopic surveillance at comparable frequencies regardless of risk. This finding highlights the need to understand barriers to risk-aligned surveillance with the goal of making it easier for clinicians to deliver it in routine practice.

## Key Points

**Question:**

Do patients with early-stage bladder cancer undergo cancer surveillance that is aligned with their risk of cancer recurrence?

**Findings:**

In this US national cohort study of patients with early-stage bladder cancer treated in 85 Department of Veterans Affairs facilities, surveillance was performed at a comparable frequency for low- and high-risk patients within 70 of 85 facilities, differing by less than 1 cystoscopy over 2 years. Across facilities, these findings were reflected in a moderately strong and significant correlation of cystoscopy frequencies for high- and low-risk patients.

**Meaning:**

Risk-aligned surveillance for early-stage bladder cancer is not widely practiced, which should alert those who care for patients with bladder cancer as well as those who care for patients with other neoplasms for which risk-aligned surveillance is recommended.

## Teaser

This cohort study assesses cystoscopy frequency for patients with low-risk vs high-risk bladder cancer treated at US Veterans Affairs facilities.

## Introduction

Guidelines for cancer surveillance routinely recommend aligning care with patients’ underlying cancer risk. Such risk-aligned surveillance entails more frequent surveillance for patients at high vs low risk of recurrence. For example, recommendations for computed tomography surveillance after treatment for kidney cancer range from every 3 to 6 months for stage II or III disease to every year for stage I disease.^1^ Similarly, after treatment for lung and prostate cancer, more frequent surveillance is recommended for patients at higher risk for recurrence.^2,3^ After treatment of colorectal adenoma, risk-aligned surveillance^4^ has already become a Medicare quality measure.^5^ Risk-aligned surveillance is likely to become even more relevant in the future, as the advent of personalized medicine increases the possibilities to predict each patient’s cancer risk.^6^

Surveillance after treatment of early-stage bladder cancer is a prime example of risk-aligned surveillance. Bladder cancer is the fourth most prevalent noncutaneous cancer in the United States.^7^ When cancer is identified, patients typically undergo transurethral resection.^8^ Based on pathologic findings, three-quarters of patients are diagnosed with early-stage disease and then undergo periodic cystoscopic surveillance with close inspection of the bladder mucosa.^9,10^ Broad consensus holds that the frequency of cystoscopic surveillance should align with each patient’s cancer risk,^11^ with risk-aligned surveillance recommended by 8 national and international panels.^8,12,13,14,15,16,17,18^ Specifically, low-risk patients are recommended to receive no more than 3 cystoscopy procedures during the first 2 years after diagnosis; high-risk patients should receive 6 to 8.^8,11^ Previous studies, including those using Surveillance Epidemiology and End Results Medicare data,^19^ have demonstrated that bladder cancer surveillance is often not aligned with underlying cancer risk. However, studies were limited to assessing care delivered in a fee-for-service environment and by lack of the longitudinal pathology data needed to accurately assign risk.

We assessed the extent to which risk-aligned surveillance is practiced across and within Department of Veterans Affairs (VA) facilities by classifying surveillance patterns for low- vs high-risk patients with early-stage bladder cancer. Taking advantage of national data—including longitudinal pathology data—from the largest integrated health care system in the United States,^20^ we sought to study national patterns of care and simultaneously identify local models of best-practice risk-aligned surveillance.

## Methods

Our overall goal was to compare the frequency of cystoscopic surveillance among patients with low-risk vs high-risk bladder cancer across and within all facilities that perform bladder cancer surveillance in the VA. We proceeded along the following 4 steps: (1) identification of a cohort of patients with newly diagnosed bladder cancer who underwent surveillance in the VA, (2) assessment of low- or high-risk cancer status, (3) measurement of cystoscopic surveillance, and (4) analyses focused on comparing surveillance frequency among low- and high-risk patients across and within facilities. The study was approved by the Veteran’s Institutional Review Board of Northern New England and the University of Utah Institutional Review Board and follows the Strengthening the Reporting of Observational Studies in Epidemiology (STROBE) reporting guideline. Patient informed consent was waived because use of the data involved no more than minimum risk to the privacy of patients and the research could not be practically conducted without waiving informed consent.

### Cohort Identification 

**Figure 1.**
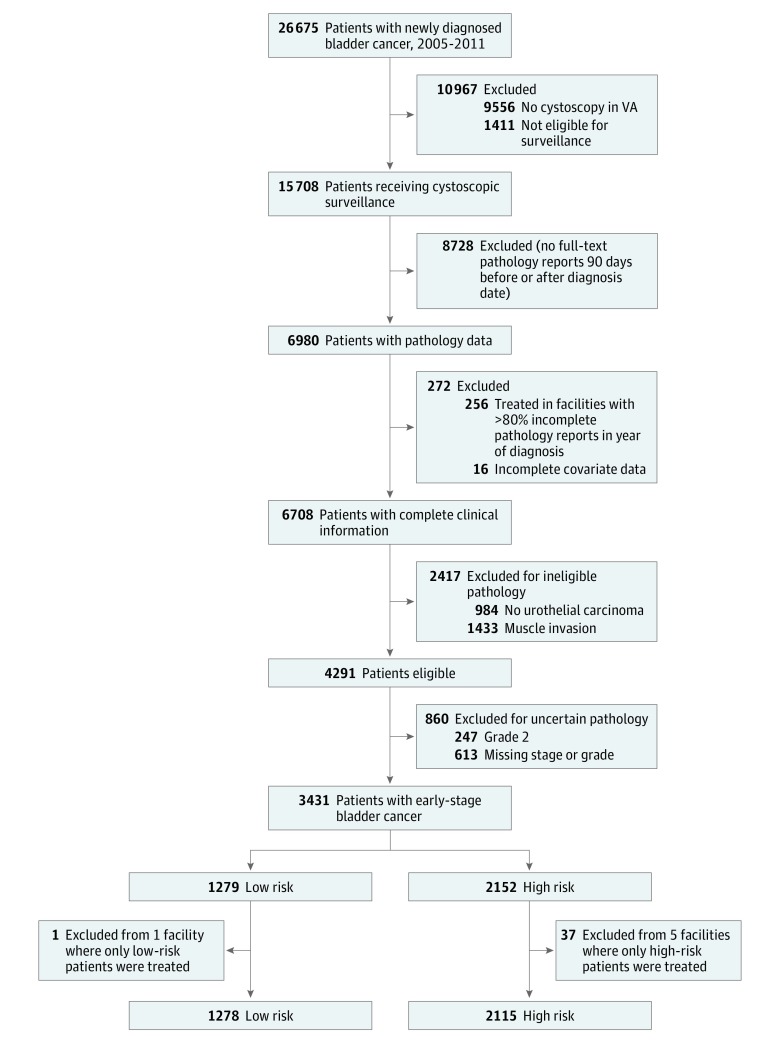
Flow Diagram of Cohort Formation The final cohort comprised 1278 patients with low-risk bladder cancer and 2115 patients with high-risk bladder cancer. VA indicates Department of Veterans Affairs.

We used VA and Medicare administrative data as well as full-text pathology data supplemented by data abstracted by VA tumor registrars, as previously described and validated,^21^ to identify all patients older than 65 years who were newly diagnosed with bladder cancer between January 1, 2005, and December 31, 2011. The diagnosis date was assigned using a previously validated claims algorithm.^21^ Next, we excluded those who underwent no cystoscopic surveillance in the VA, those not eligible for cystoscopic surveillance (based on VA or Medicare evidence of cystectomy or radiotherapy within 6 months after diagnosis), and those without pathology data around the diagnosis date (Figure 1).

Because our focus was on facility-level patterns of care, we excluded patients who were treated in facilities with a substantial proportion (>80%) of missing pathology reports (256 patients from 39 facilities). After excluding patients with incomplete covariate data (n = 16), 6708 patients with complete clinical information remained. Of these, 2417 had ineligible pathology (no urothelial carcinoma or muscle invasion indicating non–early-stage disease) and thus were not eligible for cystoscopic surveillance. Eight hundred sixty patients had uncertain pathology (grade 2 or missing grade or stage, making classification into low-risk or high-risk impossible), leaving 3431 patients with early-stage bladder cancer from 91 facilities (Figure 1).

### Assessment of Risk

Patients with early-stage bladder cancer can be stratified into low- or high-risk categories based on the pathology at the time of diagnosis. Using the European Association of Urology risk-stratification guidelines,^22^ we defined low-risk cancer status as a primary low-grade noninvasive urothelial carcinoma and high-risk cancer status as a urothelial carcinoma that was either high-grade noninvasive, invasive into the lamina propria (T1), or associated with carcinoma in situ. We operationalized these definitions using data extracted from full-text pathology reports via validated natural language processing algorithms.^23^ We used pathology reports dated 90 days before to 90 days after the diagnosis date. Because of our focus on comparing cystoscopic surveillance for low- vs high-risk patients across and within facilities, we excluded 38 patients from 6 facilities where either only low- or high-risk patients were treated. Thus, 1278 low-risk and 2115 high-risk patients from 85 facilities remained for analyses (Figure 1).

### Measuring Cystoscopic Surveillance 

We used *Common Procedural Terminology* and *International Classification of Diseases, Ninth Revision,* procedure codes to identify cystoscopy procedures during the follow-up period.^21,24^ The follow-up period started with the bladder cancer diagnosis date and ended with cancer recurrence, death, date of cystectomy or radiotherapy, date of last VA contact, or end of study (December 31, 2014), whichever occurred first. We followed patients only until they had a cancer recurrence, because a recurrence increases risk for further recurrences and thus changes a patient’s cancer risk status.^22^ We ascertained cancer recurrences using data extracted from full-text pathology reports via the validated natural language processing algorithms.^23^ Next, we enumerated the number of cystoscopy procedures each patient underwent during the follow-up period. We only counted those cystoscopy procedures that occurred at least 30 days following a previous procedure,^25^ because procedure codes occurring in close proximity to each other are unlikely to indicate routine surveillance (eg, cystoscopy with biopsy following shortly after a surveillance cystoscopy) and may sometimes even refer to the same procedure. The majority of patients (86.6% [2937 of 3393]) received cystoscopy procedures at only 1 facility; the remaining patients were assigned to the facility where they received the plurality of their cystoscopy procedures.

### Statistical Analysis

Data management was performed from December 2015 to February 2017. Data analyses were performed from March 2017 to April 2018. Descriptive statistics were used to describe both patient and facility characteristics. Patient characteristics included age at the time of bladder cancer diagnosis, sex, race, year of diagnosis, and comorbidity using the enhanced Elixhauser index.^26^ Facility characteristics were obtained from the Veterans Health Administration’s Support Service Center and included number of hospital beds, unique patients per year, urology outpatient visits per year, urologist full-time equivalents, rural location, and academic affiliation.^27^

Negative binomial multilevel models were used to calculate the adjusted cystoscopy frequency for each facility. The outcome was the patient-level number of cystoscopy procedures during the follow-up period. Models contained a random intercept for facility. They were adjusted for age (≥80 years) and comorbidity (>3 comorbidities) to account for differences across facilities in age and comorbidity, which might independently inform surveillance frequency. To account for time trends, we adjusted for year of diagnosis. We also included an indicator variable to denote whether patients were followed for longer than 2 years, because guideline recommendations specify that frequency of follow-up can be decreased at that point.^18,28^ The logarithm of length of follow-up was included as an offset.

Separate models were fit for low- and high-risk patients. From these models, we then calculated the frequency of cystoscopic surveillance for each facility with corresponding 95% confidence intervals for an average low- or high-risk patient diagnosed in 2011 using empirical Bayes estimation.^29^ This approach accounted for differences in the reliability of estimated facility-level cystoscopy frequencies due to differences in sample size by shrinking the estimates of facilities with a small number of patients closer to the overall mean.^29^

To assess the strength of correlation between cystoscopy frequencies for low- and high-risk patients across facilities, we calculated the Pearson correlation coefficient. We determined which facilities, on average, performed at least 1 cystoscopy more over 2 years for high-risk than for low-risk patients. We also assessed whether surveillance frequency was statistically significantly different within each facility for high- vs low-risk patients. For this, a separate negative binomial model for each facility was fit, with the main exposure being high- vs low-risk cancer status while adjusting for the same covariates.

### Sensitivity Analysis

We performed 2 sensitivity analyses. First, we performed a simpler analysis to address the potential concern that the multivariable multilevel modeling, adjustment, and shrinkage may have affected our findings.^29^ In this analysis, we calculated the number of cystoscopies performed per 2 years for each patient. We only included patients who had at least 1 year of follow-up and followed them for up to 2 years after diagnosis. This was done to limit the effect of very short or very long follow-up on the calculated number of cystoscopies per 2 years. Next, we limited this sensitivity analysis to facilities that had at least 10 low-risk and 10 high-risk patients in the data set (618 low-risk and 709 high-risk patients across 33 facilities). For each facility, we calculated the unadjusted mean frequency of cystoscopy for low- and high-risk patients. Strength of correlation was assessed between these frequencies using the Pearson correlation coefficient.

Second, to determine whether reliance on pathology data extracted via natural language processing affected our results, the main analyses were repeated using data abstracted by tumor registrars to characterize cancer risk rather than the natural language processing results. This sensitivity analysis included 1290 low-risk and 3987 high-risk patients. All analyses were performed using SAS statistical software version 9.4 (SAS Institute Inc) and Stata MP statistical software version 15 (StataCorp LLC). Statistical significance was set at 2-sided α < .05 for all analyses.

## Results

We included 1278 low-risk and 2115 high-risk patients with a median (interquartile range [IQR]) age of 77 (71-82) years; 99% (3368 of 3393) were male. Patients received care across 85 VA facilities from 45 states, the District of Columbia, and Puerto Rico. Each facility contributed a mean (range) of 40 (3-150) patients. 1237 of 3393 patients (36.5%) were aged 80 years or older, and 670 of 3393 (19.8%) had more than 3 comorbidities (Table 1). Facilities had a median (IQR) of 135 (81-218) hospital beds, few were in rural locations, and most had an academic affiliation (Table 2). The median (IQR) proportion of low-risk patients among the facilities was 37% (27%-50%).

**Table 1.** Demographic Characteristics of Patients^a^

Characteristic	No. (%)	
	Low Risk (n = 1278)	High Risk (n = 2115)
Age, median (IQR), y	76 (71-81)	77 (72-83)
≥80 y	412 (32.2)	825 (39.0)
Male	1265 (99.4)	2103 (99.0)
Race		
White	1079 (84.4)	1745 (82.5)
Black	88 (6.9)	157 (7.4)
Asian	9 (0.7)	19 (0.9)
Hispanic	18 (1.4)	39 (1.8)
Native American	11 (0.9)	9 (0.4)
Unknown	73 (5.7)	146 (6.9)
Comorbidities, No.		
0-3	1018 (79.7)	1705 (80.6)
>3	260 (20.3)	410 (19.4)
Year of diagnosis		
2005	119 (9.3)	198 (9.4)
2006	131 (10.3)	243 (11.5)
2007	160 (12.5)	282 (13.3)
2008	202 (15.8)	331 (15.7)
2009	217 (17.0)	347 (16.4)
2010	228 (17.8)	390 (18.4)
2011	221 (17.3)	324 (15.3)
Bladder cancer stage^b^		
Ta (noninvasive)	1278 (100.0)	754 (35.7)
T1 (invasive or suspected invasion and superficial invasion depth)	0	1267 (59.9)
Carcinoma in situ only	0	94 (4.4)
Carcinoma in situ^b^	0	417 (19.7)
Bladder cancer grade^b^		
Low	1278 (100.0)	235 (11.1)
High	0	1880 (88.9)

Abbreviation: IQR, interquartile range.

^a^ Based on full-text pathology risk assignment.

^b^ Derived via validated natural language processing algorithms.^23^

**Table 2.** Characteristics of the 85 Facilities Included

Characteristic^a^	Value
Hospital beds, median (IQR), No.	135 (81-218)
Unique patients per year, median (IQR), No.	44 220 (32 915-57 776)
Urology outpatient visits per year, median (IQR), No.	2722 (1881-3717)
Urologist full-time equivalents, median (IQR), No.	1.7 (1.1-2.6)
Rural facility, No. (%)	8 (9.4)
Academic affiliation, No. (%)	72 (92.9)

Abbreviation: IQR, interquartile range.

^a^ Characteristics were obtained from the Veterans Health Administration's Support Service Center.

Patients with low-risk cancer underwent a mean (SD) of 5.3 (3.4) cystoscopy procedures during a median (IQR) follow-up of 2.6 (0.9-4.7) years. As expected, patients with high-risk cancer had shorter follow-up periods due to a higher recurrence rate (median [IQR], 1.2 [0.6-3.7] years) during which they underwent a mean (SD) of 4.7 (3.7) cystoscopy procedures.

**Figure 2.**
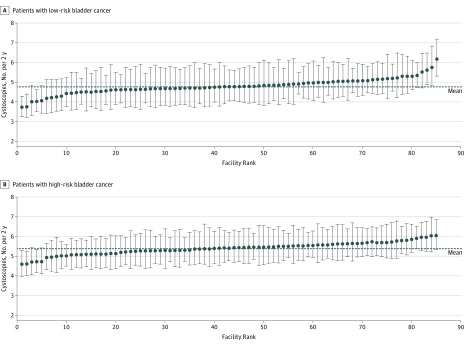
Facility-Level Variation in Adjusted Frequency of Cystoscopy Procedures for Low-Risk and High-Risk Patients Facilities are ranked from lowest frequency to highest frequency of cystoscopy for patients with low-risk (A) and high-risk (B) bladder cancer. The mean frequency across all facilities is indicated on the y-axis. Error bars indicate 95% confidence intervals. Frequency of cystoscopy was adjusted for age, comorbidity, year of diagnosis, and length of follow-up.

Across all facilities, the adjusted frequency of surveillance cystoscopy ranged from 3.7 to 6.2 (mean, 4.8) procedures over 2 years for low-risk patients (Figure 2A) and from 4.6 to 6.0 (mean, 5.4) procedures over 2 years for high-risk patients (Figure 2B). For both low-risk and high-risk patients, overlap of 95% confidence intervals around adjusted cystoscopy frequencies suggested that a common mean frequency could be representative of most facilities (Figure 2).

**Figure 3.**
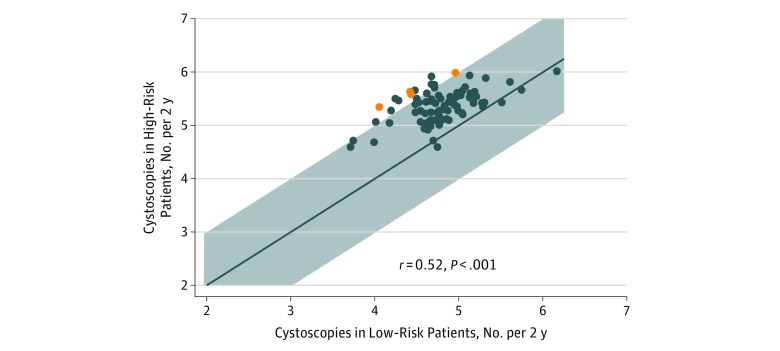
Facility-Level Correlation of Cystoscopy Frequency Between Low-Risk and High-Risk Patients Each dot represents 1 facility. The line represents the same cystoscopy frequency for low-risk patients (x-axis) and high-risk patients (y-axis). The shaded area represents facilities where low-risk and high-risk patients undergo cystoscopy at comparable rates (ie, absolute difference of less than 1 cystoscopy over 2 years). Orange dots represent facilities with a statistically significantly higher frequency for high-risk vs low-risk patients.

Within facilities, differences in cystoscopy frequency for high- vs low-risk patients were small (mean [range], 0.6 more over 2 years [0.15 fewer to 1.3 more]) (Figure 3). At most facilities (70 of 85), cystoscopy was performed at a similar frequency for high- and low-risk patients, with a difference of less than 1 cystoscopy over 2 years. At the remaining 15 facilities, there appeared to be more of a distinction between high- and low-risk patients, with a difference surpassing 1 cystoscopy over 2 years (range, 1.0-1.3 cystoscopies more over 2 years for high- vs low-risk patients). However, we found a statistically significantly higher cystoscopy frequency for high- vs low-risk patients only within 4 of the 85 facilities. Across all of the 85 facilities, these findings were reflected in a moderately strong correlation of cystoscopy frequencies for high-risk and low-risk patients (*r* = 0.52; *P* < .001) (Figure 3).

We performed sensitivity analyses using unadjusted data to characterize cystoscopy frequency for high- and low-risk patients at each facility. Again, cystoscopy frequency was moderately strongly correlated (*r* = 0.45; *P* = .008) and cystoscopy was performed at a similar frequency for high- and low-risk patients at most facilities (28 of the 33 included) (eFigure in Supplement 1). In a second sensitivity analysis, we used data abstracted by tumor registrars instead of full-text pathology data to characterize risk. Results from these analyses were not substantially different from those of the main analyses; thus, only the latter are presented.

## Discussion

We found that across the national VA integrated health care network, patients with high-risk bladder cancer undergo cystoscopic surveillance at comparable frequency to those with low-risk cancer, with few exceptions. At only 4 of 85 facilities was cystoscopic surveillance frequency significantly higher for high-risk patients than for low-risk patients, and even at these facilities high-risk patients underwent on average only approximately 1 cystoscopy more over the course of 2 years. This difference is much smaller than would be expected based on guideline recommendations, which recommend 6 to 8 cystoscopies over 2 years for high-risk patients and no more than 3 cystoscopies over 2 years for low-risk patients.^11^ Thus, we were not able to identify facilities that clearly exhibit local models of best practice risk-aligned care, although it may be possible to learn from facilities that appear to distinguish between low- and high-risk patients in their surveillance practices.

This is the first study, to our knowledge, to examine whether risk-aligned cancer surveillance is performed in a national health system. As 1 of many cancers for which ongoing surveillance is routinely recommended, early-stage bladder cancer serves as a useful paradigm for assessing surveillance practices because of its high prevalence^7^ and because surveillance with cystoscopy is identifiable using administrative data. Our findings demonstrate that facilities with appropriately high surveillance rates for high-risk cancers also have inappropriately high surveillance rates for low-risk cancers and vice versa. They highlight how challenging it can be to routinely incorporate underlying cancer risk into cancer surveillance practice and suggest non–cancer-related factors may be driving surveillance rates, regardless of underlying risk of recurrence and progression.

Prior work has examined the use of surveillance colonoscopy among patients diagnosed with colorectal adenoma. Similar to our findings, the frequency of surveillance colonoscopy was not aligned with the risk for progression to advanced lesions.^30^ Another example of patients with cancer not receiving risk-aligned care is imaging among low- and high-risk patients recently diagnosed with prostate or breast cancer.^31,32^ Diagnostic imaging is appropriate among high-risk patients and inappropriate among low-risk patients. Similar to our findings regarding risk-aligned cancer surveillance, appropriate and inappropriate imaging rates were closely correlated.^31,32^ Furthermore, efforts to decrease inappropriate imaging for patients with low-risk prostate cancer have produced the unintended consequence of decreasing appropriate imaging for high-risk patients.^33^ Taken together, these findings highlight the need to develop strategies that make it easier for physicians to deliver cancer care that is aligned with underlying cancer risk.

### Limitations

Although the present study is informed by national data and our findings reflect care received across a large number of distinct facilities, it is not without limitations. First, our data are from the VA and thus generalizability to other settings may be limited. However, previous studies of surveillance of early-stage bladder cancer using Surveillance Epidemiology and End Results Medicare data have also found that risk of cancer recurrence had little impact on the care patients receive.^19^ Second, the number of patients within each facility affected the power to detect any statistically significant differences in surveillance frequency between low-risk and high-risk patients. Thus, we calculated the shrunken surveillance frequency for each facility in our main analyses—an approach that adjusted for the reduced reliability of estimates in smaller facilities.^29^ Third, we acknowledge certain limitations of ascertaining cancer risk. While we used a validated natural language processing engine to extract information from pathology reports,^23^ certain characteristics that may contribute to an increased risk of cancer recurrence among low-risk patients (such as multifocality and large tumor size, classified as intermediate risk in the European Association of Urology risk-stratification guidelines^22^) could not be assessed. Thus, among low-risk patients, there are likely subgroups of patients who are at lower and somewhat higher risk of cancer recurrence. However, as all low-risk patients had low-grade disease, they are all still at substantially lower risk than the high-risk patients. Finally, the analysis examining differences in surveillance frequency between low- and high-risk patients is predicated on recommendations for risk-aligned surveillance that are based on lower level evidence.^11^ Nevertheless, 8 panels reviewing current evidence have recommended risk-aligned surveillance,^8,12,13,14,15,16,17,18^ which allows for timely detection of progression to muscle-invasive cancer among high-risk patients while sparing low-risk patients unnecessary procedures. Timely detection is important because delays in diagnosis of muscle-invasive cancer are associated with increased mortality.^34^ Avoiding unnecessary procedures is relevant for patients as they lead to more anxiety, discomfort, and costs.^35,36^

### Strengths

These limitations notwithstanding, our study has important strengths. Because we had access to full-text pathology reports, this is the first population-based study, to our knowledge, on early-stage bladder cancer surveillance in which cancer recurrence could be ascertained. Because recurrence elevates patients’ future risk of further recurrence and thus influences further surveillance, we censored follow-up at the time of cancer recurrence, which has not been possible using other population-based data sets. Second, we used multilevel modeling to account for differences in reliability of facility-level estimates due to differences in the number of patients per facility. Third, to address potential concerns that the use of complex modeling or data obtained via natural language processing affected the validity of our findings, we performed sensitivity analyses using unadjusted data and data abstracted by tumor registrars, which supported our main findings.

Our study is the first to assess risk-aligned bladder cancer surveillance in the VA, which has important implications for health policy and future research. Some may argue that financial incentives in a fee-for-service environment are an important factor contributing to more cystoscopy procedures than recommended. However, the VA is a capitated health care system in which such financial incentives do not exist, and we still find substantial overuse of cystoscopy among low-risk patients. In fact, we recently performed patient-level analyses to examine the extent of overuse and found that the number of cystoscopy procedures among low-risk patients is about double what it should be.^37^ Thus, other factors are likely important contributors to the lack of risk-aligned surveillance and may include limited clinician knowledge of the guidelines, clinicians’ habit to continue what they have always done, and complexity and variability of risk stratification across different guidelines.^11^ Future work should focus on understanding the barriers to risk-aligned surveillance and on developing strategies to improve surveillance. These strategies should facilitate providers’ ability to deliver risk-aligned surveillance, thus reducing adverse consequences of overuse of surveillance among low-risk patients and of underuse of surveillance among high-risk patients.

## Conclusions

In conclusion, our study highlights that risk-aligned surveillance for early-stage bladder cancer is not widely practiced. On the contrary, patients with high- and low-risk cancer undergo surveillance at comparable frequency, despite recommendations that high-risk patients warrant surveillance at least twice as often.^11^ Our findings should alert those who care for patients with bladder cancer and those who care for patients with other neoplasms for which risk-aligned surveillance is recommended. While risk factors, natural history, and tumor-specific characteristics differ across neoplasms, the challenges clinicians face to align surveillance with underlying cancer risk are likely similar.

## Notes

Supplement 1. **eFigure.** Sensitivity Analysis Examining Facility-Level Correlation of Cystoscopy Frequency Between Low-Risk (x-axis) and High-Risk (y-axis) Patients

Supplement 2. **Data Sharing Statement**
