# Supplementary material for: Extent of Risk-Aligned Surveillance for Cancer Recurrence Among Patients With Early-Stage Bladder Cancer
Source: JAMA Netw Open. 2018 Sep 28;1(5):e183442. doi: 10.1001/jamanetworkopen.2018.3442 (PMC6241521; doi:10.1001/jamanetworkopen.2018.3442)
Supplement: Supplement 1. — eFigure. Sensitivity Analysis Examining Facility-Level Correlation of Cystoscopy Frequency Between Low-Risk (x-axis) and High-Risk (y-axis) Patients [file jamanetwopen-1-e183442-s001.pdf]

Supplementary Online Content

Schroeck FR, Lynch KE, Chang Jw, et al. Extent of Risk-Aligned Surveillance for Cancer Recurrence Among Patients With Early-Stage Bladder Cancer. *JAMA Netw Open.* 2018;1(5):e183442. doi:10.1001/jamanetworkopen.2018.3442

eFigure. Sensitivity Analysis Examining Facility-Level Correlation of Cystoscopy Frequency Between Low-Risk (x-axis) and High-Risk (y-axis) Patients

This supplementary material has been provided by the authors to give readers additional information about their work.

© 2018 Schroeck FR et al. *JAMA Network Open.*

eFigure. Sensitivity analysis examining facility-level correlation of cystoscopy frequency between low-risk (x-axis) and high-risk (y-axis) patients. The data presented here is based on the raw data, without adjusting for co-variates or reliability. Only facilities with at least 10 low-risk and 10 high-risk patients were included. The line represents the same cystoscopy frequency for low- and high-risk patients. The shaded area represents facilities where low-risk and high-risk patients undergo cystoscopy at comparable rates (*i.e.,* absolute difference of less than 1 cystoscopy over 2 years). Green dots represent facilities with a statistically significantly higher frequency for high-risk versus low-risk patients. Cystos = cystoscopies.

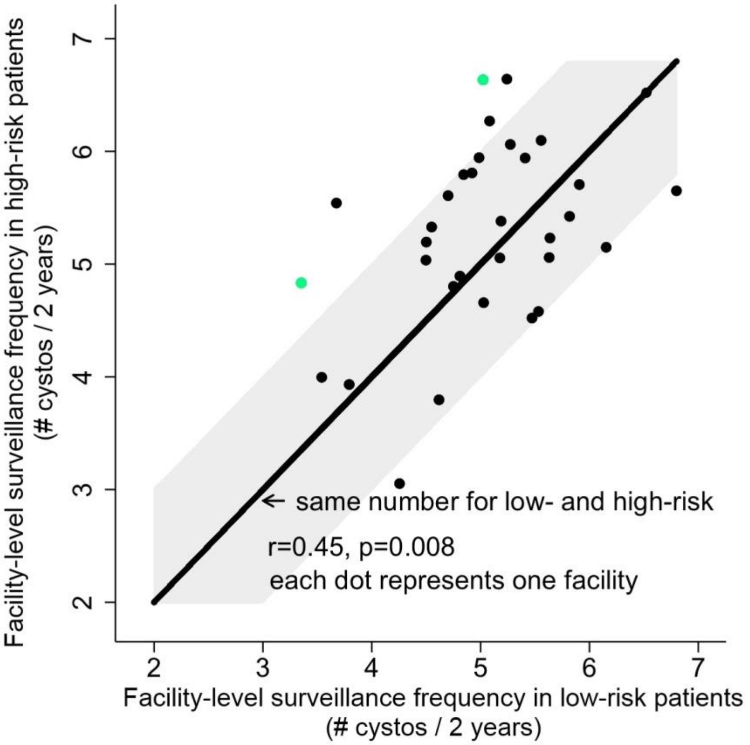

© 2018 Schroeck FR et al. *JAMA Network Open.*
