# Supplementary material for: Extent of Risk-Aligned Surveillance for Cancer Recurrence Among Patients With Early-Stage Bladder Cancer
Source: JAMA Netw Open. 2018 Sep 28;1(5):e183442. doi: 10.1001/jamanetworkopen.2018.3442 (PMC6241521; doi:10.1001/jamanetworkopen.2018.3442)
Supplement: Supplement 2. — Data Sharing Statement [file jamanetwopen-1-e183442-s002.pdf]

Data Sharing Statement

Data
Data available: No
